# Dr. Sam

**Published:** 1895-10

**Authors:** Eugene Field


### DR. SAM.

Down in the old French quarter
(Just out of Rampart street)
I went my way
Unto the quaint retreat
Where lives the Hoodoo doctor,
By some esteemed a sham—
Yet I'll declare there's none elsewhere
So skilled as Dr. Sam.
With claws of a devilled crawfish,
The juice of a prickly-prune,
And the quivering dew
From a yarb that grew
In the light of a midnight moon!

I never should have known him
But for the colored folk
That here obtain
And ne'er in vain
That wizzard's arts invoke;
For when the Eye that's Evil
Would him and his'n damn,
The negro's grief gets quick relief
Of Hoodoo-Doctor Sam!
With the caul of an alligator,
With the plume of an unborn loon,
And the poison wrung
From a serpent tongue
By the light of a midnight moon!

In all neurotic ailments
I hear that he excels
And he insures
Immediate cures
Of weird, uncanny spells;

The most unruly patient
  Gets docile as a lamb
And is freed from ill by the potent skill
  Of Hoodoo-Doctor Sam!
    Feathers of strangled chickens,
      Moss from the dank lagoon,
        And plasters wet
        With a spider's sweat
      In the light of a midnight moon!

They say when nights are grewsome
  And hours are, oh! so late,
    Old Sam steals out
    And hunts about
  For charms that hoodoos hate!
That from the moaning river
  And from the haunted glen
He siliently brings what eerie things
  Give peace to hoodooed men—
    The tongue of a piebald possum,
      The tooth of a senile coon,
The buzzard's breath that pants for death,
      And the film that lies
      On a lizard's eyes—
  'Neath the light of a midnight moon!

—*Eugene Field in the Medical Examiner.*

